# Q2DSTD NMR deciphers epitope-mapping variability for peptide recognition of integrin αvβ6†
†Electronic supplementary information (ESI) available. See DOI: 10.1039/c5ob01237f



**DOI:** 10.1039/c5ob01237f

**Published:** 2015-06-29

**Authors:** Jessica L. Sorge, Jane L. Wagstaff, Michelle L. Rowe, Richard A. Williamson, Mark J. Howard

**Affiliations:** a School of Biosciences , University of Kent , Canterbury , Kent , UK . Email: m.j.howard@kent.ac.uk

## Abstract

Integrin αvβ6 is a cell surface arginine-glycine-aspartic acid (RGD)-specific heterodimeric glycoprotein that is only expressed on epithelia during processes of tissue remodelling, including cancer. The specificity and molecular nature of interactions toward this integrin are poorly understood and new insights into such processes are important to cell biologists and pharmaceutical drug discovery. This study demonstrates the application of quantitative two-dimensional saturation transfer (Q2DSTD) NMR to obtain precise details of peptide interactions with integrin αvβ6 and their correlation to specificity for the integrin. This approach highlights subtle but significant differences in ligand contact by three related 21-mer peptides: FMDV2, an αvβ6 specific peptide and DBD1 and LAP2T1 peptides that bind many αv integrins in addition to αvβ6. FMDV2 and DBD1 differ only by the cyclisation of DBD1; a process that removes αvβ6 specificity. Q2DSTD NMR demonstrates these peptides experience significantly different interactions with the integrin; FMDV contacts primarily through four residues: 6Leu, 10Leu, 12Val and 13Leu, whereas DBD1 and LAP2T1 have more widespread contacts across their sequences. Q2DSTD NMR combined two-dimensional STD with quantitation by considering the relaxation of the ligand (CRL) to provide precise ligand contact information. This study also examines the role of CRL in the Q2DSTD process and how quantitation modifies STD data and unravels epitope-mapping variability to provide precise results that differentiate interactions at the atomic level for each peptide.

## Abstract 2

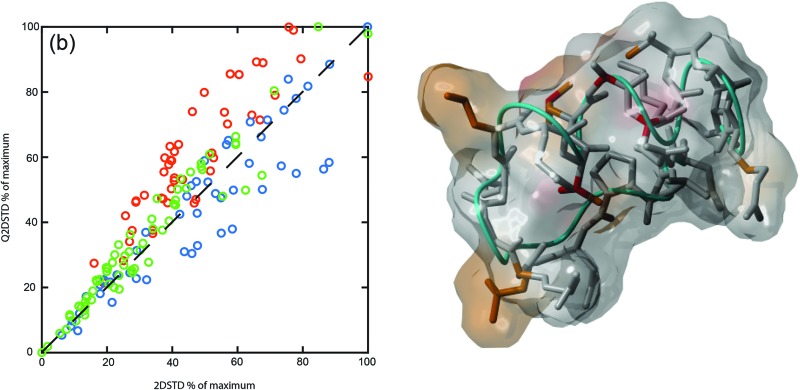
 ^1^H *T*_1_ relaxation modified 2D STD NMR reveals integrin αvβ6 molecular specificity.

## Introduction

Saturation transfer difference (STD) NMR is ideal for studying the molecular detail of target-ligand recognition.^1–3^ This study reports the use of Q2DSTD (Quantitative 2D saturation transfer difference); a combination of two-dimensional (2D) ^13^C-edited STD NMR group epitope mapping^2^ with added consideration of the relaxation of the ligand (GEM-CRL),^4^ to distinguish subtle contact differences between three peptides with diverse specificities toward integrin αvβ6. Group epitope mapping (GEM) is an approach where the relative intensities of STD signals provide information regarding the proximity of each ligand chemical group to the protein-target when it binds.^2^ This approach is powerful because it informs on the closest and most significant molecular contacts that relate to ligand binding-mode and significant molecular interactions. Q2DSTD has the power to decipher subtle contact differences that are difficult to distinguish using standard STD and GEM methods. 2D heteronuclear editing is required to unravel the large number of degenerate ^1^H chemical shifts that are problematic from peptide samples containing several similar or identical amino acids. Considering the relaxation of the ligand involves subsequent data modification where ^1^H longitudinal relaxation (T_1_) relaxation adjusts the STD contact result that can be distorted by structural anisotropy, non-specific aggregation and amphipathicity. The expected variability in ^1^H *T*_1_ across each peptide sequence is influenced by amino acid composition and nascent structure and cannot be easily predicted. This study demonstrates advantages of *T*_1_ adjusted STD data for peptides to ascertain precise contact information to understand contacts responsible for specificity. It is most striking because we demonstrate QSTD contacts vary across three biologically distinct peptides that are all 21-mers of similar molecular weight. Only through analysis of precise quantitative STD data will significant contacts relevant to ligand specificity be identified.

Integrins are heterodimeric glycoproteins composed of non-covalently linked α and β subunits^5^ that dynamically translate extracellular matrix cues into intracellular responses to modulate cell proliferation, survival, migration and invasion.^6^ Integrin αvβ6 is only expressed on epithelia during processes of tissue remodeling such as wound healing, inflammation and cancer.^7,8^ Integrin αvβ6 has been identified as conveying a pro-invasive and aggressive phenotype when overexpressed on cancer cells;^8–11^ it is an emerging clinical target as survival from cancers is reduced significantly if high levels of this integrin are expressed.^12^ The real challenge revolves around finding ligands that bind only to αvβ6 that can be developed into medical agents and is complicated by αvβ6 being a arginine-glycine-aspartic acid (RGD) receptor class integrin that include αvβ3, αvβ5, αvβ8, α8β1, α5β1 and αIIbβ3; all of which bind molecules that contain the RGD motif. STD NMR has previously been used to study integrin αvβ6-peptide recognition.^13–15^ 1D ^1^H and 2D ^13^C-edited STD NMR validated the affinity toward αvβ6 within peptides containing a turn-helix and a primary interacting extended RGDLXXL/I motif. FMDV2 is the only RGD-peptide ligand known to bind exclusively to αvβ6, but peptides DBD1 and LAP2T1 exhibit recognition to αvβ6 and a variety of other αv integrins including αvβ5, αvβ6 and αvβ8.^16^

The Q2DSTD approach was used to highlight molecular differences in peptide recognition that could be attributed to FMDV2's specificity for αvβ6. What is equally interesting is the comparison between FMDV2 and DBD1; these peptides are related in sequence but structurally distinct through the disulfide-bond cyclisation of DBD1. The fact that FMDV2 is specific to αvβ6 but DBD1 is not supports the peptide–integrin interaction as extremely sensitive to both sequence and structure.^16^ The three peptides used in this study were:FMDV2 → NAVPNLRGDLQVLAQKVART-HslDBD1 → EKCPNLRGDLQVLAQKVCRT-HslLAP2T1 → GFTTGRRGDLATIHGLNRPF-Hslwhere Hsl is a C-terminal homoserine lactone produced as a result of recombinant expression and purification.^17^ LAP2T1 is a modified latency associated peptide of TGF-β1 with a M16L mutation to enable recombinant production.^16,17^

## Results and discussion

**Fig. 1:**
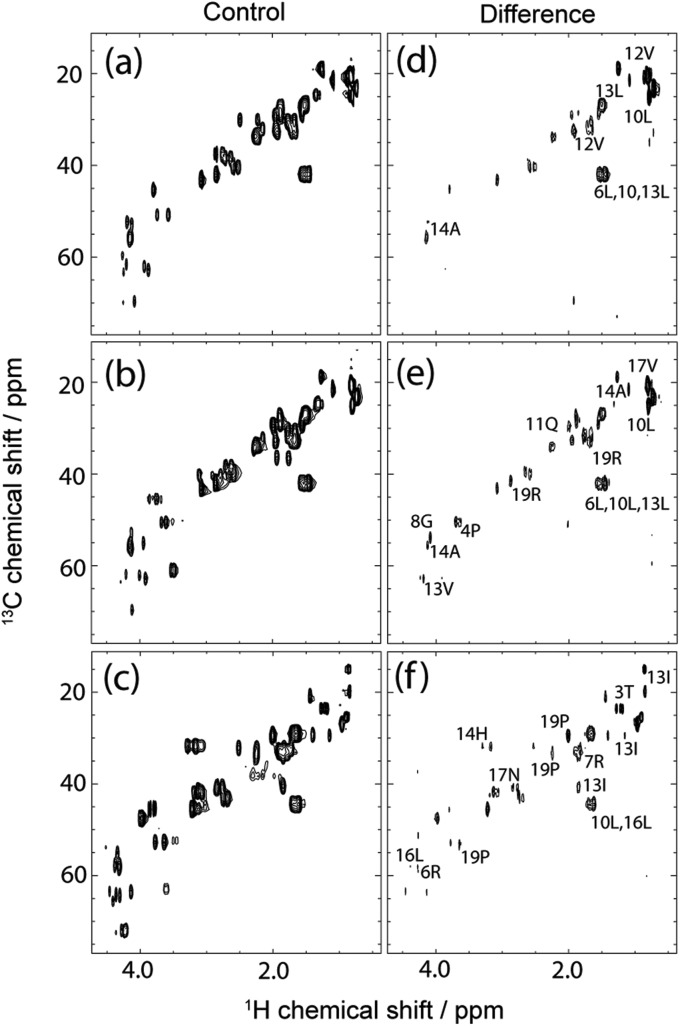
^13^C,^1^H-STD HSQC spectra of the aliphatic regions for 1 mM ligands FMDV2 (a,d), DBD1 (b,e,) and LAP2T1 (c,f) with 19 μM integrin αvβ6. Key resonances are labelled with residue number and data were acquired at 14.1 T and 10 °C.

^13^C-edited 2D STD NMR data, from samples containing identical concentrations of peptide and integrin, displayed the interaction of each peptide. The spectra labelled with key contact resonances shown in Fig. 1 are in agreement with those observed from previous studies^13,14^ and peptide ^1^H and ^13^C assignments are tabulated in the ESI.† However, these previous studies provided no STD adjustment for ligand relaxation to provide quantitative contact analysis.

**Fig. 2:**
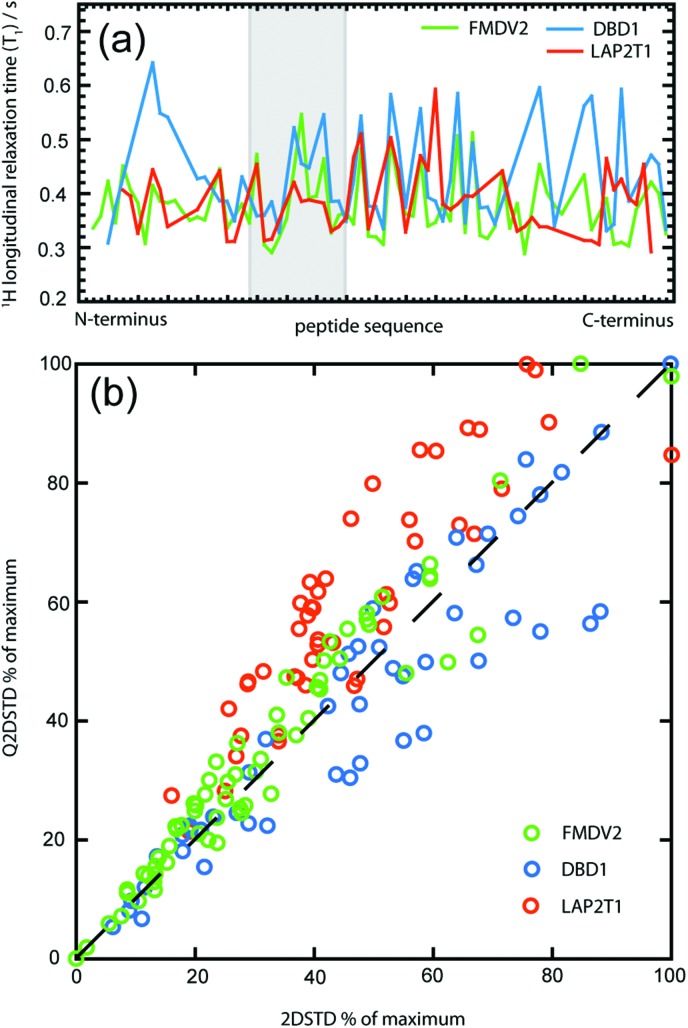
The variability of ^1^H longitudinal relaxation time (*T*_1_) across all peptide sequences (a) with RGD motif data greyed out. Correlation graph highlighting differences in Q2DSTD% *versus* 2DSTD% for each measured data point in all peptides (b). FMDV2 data is shown in green, DBD1 data in blue and LAP2T1 data in red.

^1^H *T*_1_ times measured using 2D ^13^C-edited inversion recovery varied between 0.29 and 0.64 s across all peptides (Fig. 2a) and displayed no correlation between sequences. The pulse sequence for 2D inversion recovery is shown in the ESI.† DBD1 provided the greatest variation in ^1^H *T*_1_ across the sequence that most likely reflects the cyclic configuration of this peptide that contributes structural anisotropy and subsequent *T*_1_ variability. The influence of ^1^H *T*_1_ on Q2DSTD is demonstrated in Fig. 2b, where the relative to maximum 2DSTD and Q2DSTD signals are plotted for all measured ^1^H in each peptide to demonstrate deviations caused by *T*_1_ relaxation. Proton longitudinal relaxation provides an average amendment of ±8.2% from non-adjusted 2DSTD_amp_% (standard deviation of 7.8%) but for the extremes 2DSTD_amp_% was modified by up to ±30%. Fig. 2b also illustrates the most significant differences occurring at higher Q2DSTD_amp_% values as well as LAP2T1 values being generally underestimated in contrast to DBD1 values that were overestimated. This supports the expected dependence of precision in STD being dependent on ^1^H *T*_1_ values from the ligand. Also, the sequence similar but structurally distinct FMDV2 and DBD1 peptides produce very different Q2DSTD *vs.* STD correlations and confirms the need to handle peptide STD data with GEM modification where structural differences are known to exist. Fig. 2 clearly demonstrates that different peptides of the same length can exhibit enhanced or reduced Q2DSTD signals due to variability in ^1^H *T*_1_ that subsequently influences each closest contact result at the atomic level. ^1^H *T*_1_, 2DSTD and Q2DSTD data for each peptide are fully tabulated in the ESI.†

**Fig. 3:**
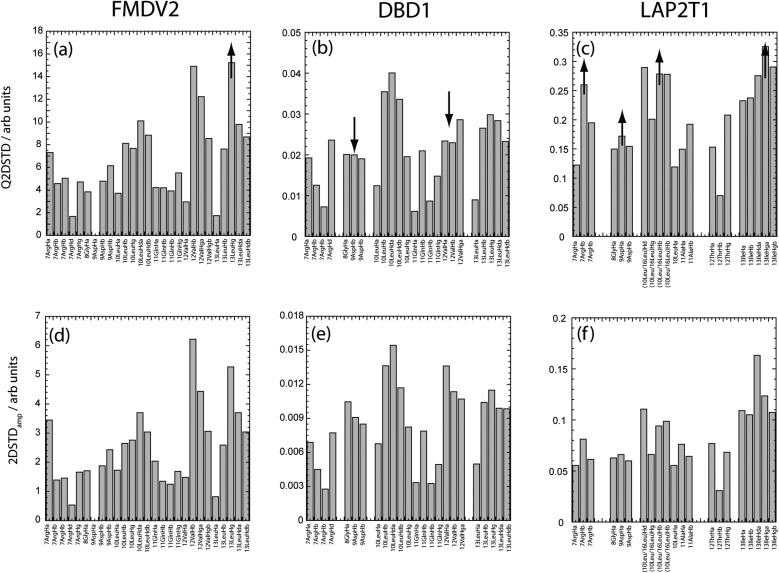
Comparison of calculated Q2DSTD_amp_ and 2DSTD_amp_ values across the RGDLXXL/I motif for FMDV2 (a,d), DBD1(b,e) and LAP2T1(c,f) following the interaction with integrin αvβ6. Changes due to quantitation are depicted with the arrows in figures (a,b,c) and demonstrate how QSTD data has elevated (↑) or suppressed (↓) non quantitative STD results across individual protons in each peptide. FMDV2 and DBD1 have identical amino acid sequences across this motif region. Gaps are included to ensure residue positions are identically placed in each graph despite missing assignments, data or sequence differences (in the case of LAP2T1).

However, detailed analysis of Q2DSTD in contrast to 2DSTD is shown in Fig. 3 where data from both approaches are shown across the RGDLXXL/I motif for each peptide. Arrows on the Q2DSTD data describe the result of *T*_1_ correction for Q2DSTD enhanced (↑) or suppressed (↓). On a global level, the 2DSTD and Q2DSTD values are significantly different for each peptide, despite each experiment being acquired identically with the same concentrations. Global STD values for FMDV2 are largest with the smallest from DBD1 with LAP2T1 approximately an order of magnitude higher than values from the cyclic peptide. This is a reflection of the relative peptide binding affinities to integrin αvβ6 that is in the order DBD1 > FMDV2 > LAP2T1 ^16^ that is corroborated from integrin αvβ6 IC_50_ data for FMDV2 and LAP2T1 of 1.2 μM and 13.8 μM respectively.^13^ Previous data^13,16^ enabled an estimate of the IC_50_ of DBD1 for αvβ6 of *ca.* 0.5 μM. STD NMR is extremely sensitive to *K*_d_ and associated off-rate^3^ where the smaller the *K*_d_, the smaller the off-rate observed. Off-rate is crucial for the detection of the ligand because it dictates the dissociation of the protein-ligand complex upon which saturation transferred to the ligand whilst bound is finally observed. If the on-rate is diffusion limited at 1 × 10^–7^ s^–1^ M^–1^, the off-rate is 0.1 s^–1^, 1.0 s^–1^, 10.0 s^–1^ for *K*_d_ values of 10 nM, 100 nM and 1 μM respectively. DBD1 has a small *K*_d_ and off-rate and produces weak STD values compared to FMDV2 that dissociates 10–100 times faster. STD data for LAP2T1 suggests this ligand may dissociate too quickly for optimal saturation transfer, hence its values are greater than DBD1 but less than FMDV2. As a result, it is likely that LAP2T1, with methionine at position 16 instead of leucine, is a weaker binder than the original LAP TGF-β1 αvβ6 peptide.^16^ These binding characteristics provide a STD and QSTD ‘sweet-spot’ that FMDV2 utilises to deliver the largest signals.

Fig. 3 confirms Q2DSTD reports different major contacts for each peptide; 13LeuHγ for FMDV2, 10LeuHδ for DBD1 and 13IleHγ for LAP2T1. The contact from 13LeuHγ in FMDV2 provided the maximum Q2DSTD signal (Fig. 3a) and elevates the significance of this contact as greater than 12ValHβ that was superior in the non-adjusted 2DSTD (Fig. 3d). This Q2DSTD result is important because it is in contrast with previous studies that identify 12Val as the most significant contact residue. The elevation of 13Leu in FMDV2 is the only major change that occurs as a result of Q2DSTD analysis but both DBD1 and LAP2T1 provide additional data modifications that are of functional interest.

In contrast to FMDV2, Q2DSTD analysis of DBD1 data delivers maxima across 10Leu Hβ and Hδ protons and suppressed 2DSTD data points from 8Gly, 9Asp and 12Val when comparing Fig. 3b with Fig. 3e. The suppression of data upon quantitation is caused by relatively long ^1^H *T*_1_ values for protons in these amino acids caused by the cyclic nature of this peptide. Suppression of 12Val signals in particular highlight the primary DBD1 contact with the integrin originating from 10Leu where Q2DSTD values *ca.* 1.3 times higher from 10Leu than 12Val or 13Leu. In contrast, FMDV2 produced Q2DSTD data that was *ca.* 1.6 times higher for 12Val and 13Leu than 10Leu. These observations enable differentiation of interactions between two similar peptides and integrin αvβ6. The disulphide bond cyclisation of DBD1 has influenced peptide primary contact with the integrin and changed specificity. FMDV2 is the only integrin αvβ6 specific peptide published to date and it is also the only peptide to show significantly strong QSTD contacts toward the C-terminus of the RGDLXXL/I motif and weaker contacts from residues between positions 7–11.

LAP2T1 Q2DSTD data also produced significant modification of data between 2DSTD and Q2DSTD (Fig. 3c or f) with Q2DSTD showing enhancements across 7ArgHβ, 8GlyHα, 10/16LeuHβ and 13IleHγ. Despite the ambiguous assignments for 10/16Leu, LAP2T1 clearly displays elevated 7Arg and 8Gly that confirms widespread major contacts across most of the RGDLXXL/I motif. Q2DSTD analysis alters the strongest amplification factor from 13IleHγ to 13IleHδ in LAP2T1 that may sound insignificant, but not to pharmacophore design.

The combined Q2DSTD results from all three peptides separate FMDV2 as only interacting with integrin αvβ6 through the C-terminus of the RGDLXXL/I motif; an observation coincident with its unique specificity for the integrin. Further analysis of Fig. 3a confirms this RGD section produces an average Q2DSTD of 4.3 compared to 15.2 for 13LeuHγ in FMDV2. Therefore the RGD Q2DSTD average is only 28% of the maximum motif signal. In contrast, the average from the same RGD regions in DBD1 and LAP2T1 rise to 43% and 52% of the maximum motif signal respectively.

**Fig. 4:**
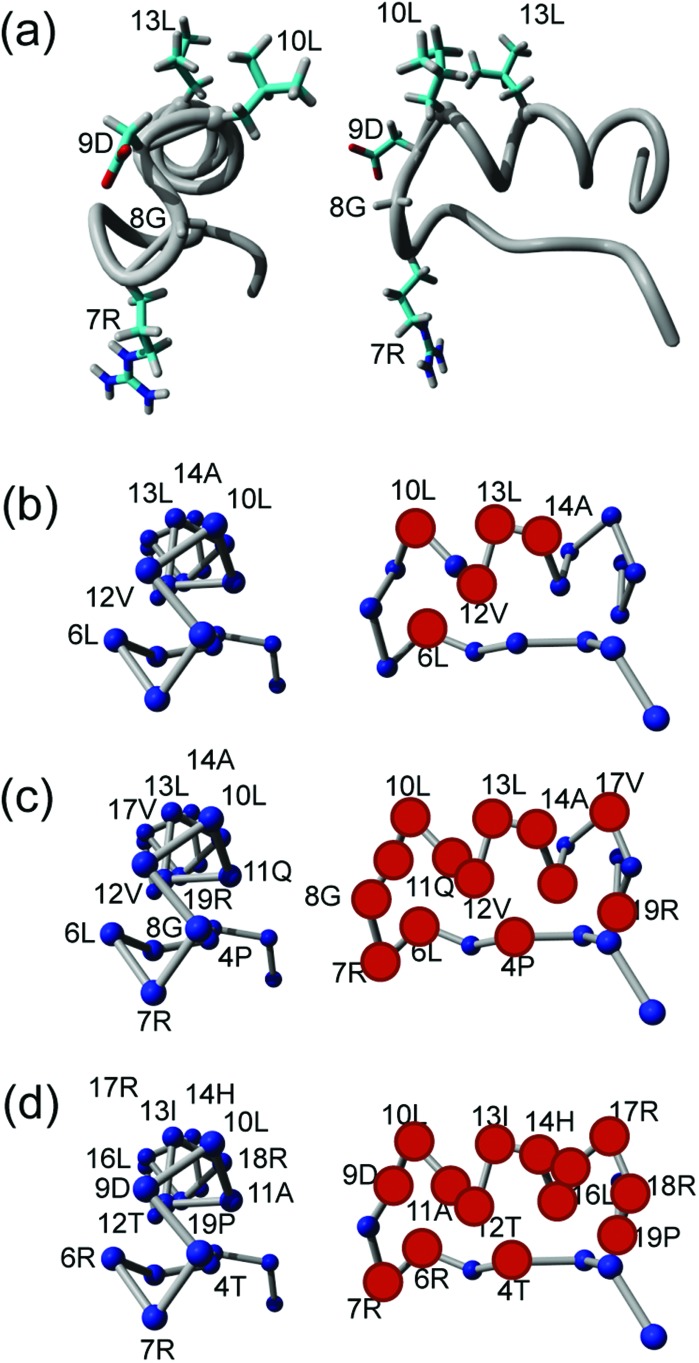
Backbone view of the FMDV2 structure with the RGDLXXL motif highlighted (a).^13,16^ Principal Q2DSTD contacts (>Q2DSTD% + 0.25*σ*) are labelled for FMDV2 (b), DBD1 (c) and LAP2T1 (d) and highlighted as large red spheres in the side on structure. This supports the wider contact by DBD1(c) and LAP2 (d) and specific contact by FMDV2 (b).

There are also advantages to viewing Q2DSTD across the entire sequence as shown in Fig. 4 with the results now reported for each peptide as percentage of their individual maximum signals. Amino acids for each peptide that contains at least one Q2DSTD% value greater than the mean Q2DSTD% + 0.25*σ* (50%) are shown as spheres to illustrate the extent of potentially significant contacts across each peptide. Using this approach Fig. 4 fully supports conclusions from Fig. 3 that FMDV2 has a smaller significant contact surface compared to DBD1 and LAP2T1. The 12Val-13Leu contact maxima from FMDV2 are distinct and easily identifiable and the ‘–KVART’ C-terminal region of this peptide produces Q2DSTD values <50%. In contrast DBD1 and LAP2T1 show values >50% in their C-terminal residues and support the hypothesis that significant contact from peptides that are not αvβ6 specific is more widespread across their sequences. The same analysis can be made regarding the N-terminal regions of DBD1 and LAP2T1. The Q2DSTD data suggests the specific nature of FMDV2 toward αvβ6 is driven by a small number of specific contacts in the peptide, with only 6Leu, 10Leu, 12Val and 13Leu registering relative Q2DSTD% values within 50% of maximum. Equivalent residues with Q2DSTD% > 50% are widespread across the sequences of DBD1 and LAP2T1. Q2DSTD% data for the entire sequence of each peptide is shown in the ESI† and further supports a specific contact for FMDV2 and contacts over a larger proportion of both DBD1 and LAP2T1 peptides. In addition, previous STD studies noted that the RGD-motif registered weakly in FMDV2.^13,14^ This was also observed in Q2DSTD data (Fig. 3 and ESI†) and confirms the original observation was not as a manifestation of ^1^H *T*_1_ effects. FMDV2 also exhibits a uniquely weak 8GlyHα interaction that could also explain its poor specificity for other RGD-specific αv integrins. This hypothesis was alluded to in earlier 1D STD data^13^ but was previously difficult to confirm due to spectral overlap.

Using QSTD2D to compare the extended FMDV2 peptide to the disulphide bond cyclized DBD1 highlights similar patterns of interaction. However, the DBD1 peptide has a prominent (>50% QSTD_amp_) RGD interaction that is not reflected in the FMDV2 data and could explain the promiscuity of DBD1 for other αv integrins. This is most likely the result of structural or conformation changes induced by cyclisation and supports the previous observation linking the rigidity of FMDV2 to αvβ6 specificity as well as conformational exchange to the loss of specificity by DBD1.^16^ Furthermore, and whatever the effects of cyclic formation, DBD1 has functional behaviour akin to LAP2T1 and LAP2T1 was also shown to be conformationally active.^16^

Close examination of individual contacts across the RGDLXXL/I motif (Fig. 3) highlight the C-terminal motif preference for primary contact to the integrin by FMDV2. This detail informs that integrin αvβ6 specificity involves a small binding site on the integrin and that FMDV2 is capable of accessing this binding site and also suppressing its interactions with other αv integrins. This does suggest that shortening the FMDV2 sequence would create smaller peptides with equivalent properties but preliminary data suggests both αvβ6 specificity and affinity are lost upon FMDV2 truncation at either N- or C-terminus. This supports the original hypothesis that specific peptides for integrin αvβ6 require a turn-helix motif with a stabilised helical region beyond the RGDLXXL/I motif.^13^

## Conclusions

Q2DSTD is a powerful approach using isotopically enriched peptides to provide resolution, combined with quantitative correction of ligand longitudinal relaxation. This study demonstrated a valuable application of Q2DSTD NMR in providing specific contact information of similar peptides that highlight molecular specificity and recognition toward integrin αvβ6. These specific contacts from the FMDV2 peptide provide the first insight into the design of pharmacophores to create future pharmaceutical agents against this cancer integrin. In this study, the molecular features required for integrin αvβ6 specificity can be seen in Fig. 4b and need to mirror contacts made by 6Leu, 10Leu, 12Val, 13Leu and 14Ala with the primary contact point based in the vicinity of 13Leu.

## Experimental

### Recombinant peptide production

All laboratory reagents reagent grade or higher and supplied by Sigma-Aldrich unless otherwise stated. The production and purification of recombinant peptides is described below and also in our previous publications.^16,17^ FMDV2 originates from the surface GH loop motif from foot and mouth disease virus serotype O_1_ BFS capsid protein VP1.^18^ DBD1 is a variant of FMDV2 with a cyclizing disulfide bond to limit serum degradation for clinical *in vivo* use. LAP2T1 is a natural αvβ6 ligand sequence from the latency-associated peptide (LAP) of transforming growth factor-β1 (TGFβ1).^13^

The recombinant peptide production process uses the pET-31b vector in *E. coli* that utilises ketosteroid isomerase as the fusion partner with the peptide to facilitate easy extraction with minimal loss of the peptide due to proteolysis. This is achieved because the expression product is insoluble and forms inclusion bodies of the product. These inclusion bodies are easily purified, then solubilised and cleaved to yield peptide that is finally purified to >90% by HPLC. Isotopic enrichment involves the growth of *E. coli*, complete with the pET-31b plasmid encoding the peptide, in a minimal medium with uniform labelled ^13^C-glucose; the same approach used for making isotopically enriched proteins.

Sense and anti-sense oligonucleotides (MWG) were designed to encode peptide sequences for FMDV2, DBD1 and LAP2T1. Oligonucleotides were 5′ phosphorylated and designed with additional 3′ overhangs of ATG for the sense and CAT for the anti-sense sequence so that annealed DNA could be ligated into the pET31b(+) (Novagen) vector pre-cut with *AlwN* I restriction enzyme. Oligonuclotides were inserted downstream of the N-terminal fusion protein ketosteroid isomerase and upstream of a C-terminal His-tag. Ligation mixtures were transformed into competent *E. coli* DH5α cells and selected for by plating on ampicillin LB agar plates. Colonies were screened for oligonucleotide insertion by PCR or restriction digestion of purified plasmid using Xba I and Xho I restriction enzymes. Sequences of plasmids containing multiple inserts were sequenced and if correct transformed into competent *E. coli* BL21(DE3) cells ready for recombinant protein expression.

Recombinant ^13^C isotopically enriched fusion protein was expressed in minimal M9 medium at 37 °C at 200 rpm with ^15^N ammonium sulphate (Cambridge Isotopes, USA) as the sole nitrogen source. Protein expression was induced by addition of IPTG to a final concentration of 1 mM for 3–4 h when the OD600 nm of the culture was between 0.55 and 0.7. Cells were harvested by centrifugation (15 min, 6300*g*) and the cell pellet re-suspended in lysis buffer (20 mM NaH_2_PO_4_, 50 mM NaCl, pH 7.3; 10 mL per 400 mL of original culture volume) and frozen. After thawing, cell lysis was completed by the addition of lysozyme to a final concentration of 0.01 mg mL^–1^ and Triton X-100 at 0.1% v/v and incubated at RT for 20 min followed by the addition of 0.02 mg mL^–1^ DNase I and 10 mM MgCl_2_ until the viscosity of the solution was reduced followed by 2 min of pulsed sonication on ice. Insoluble fusion protein was then recovered from the total cell lysate by centrifugation (10 min, 12 000*g*) and purified by re-suspension in wash buffer (50 mM Tris-HCl, 10 mM EDTA, 0.5% Triton X-100, pH 8; 2.5 mL per 400 mL of original culture volume) and recovering by centrifugation (10 min, 12 000*g*), with this step repeated again with wash buffer and then a further two times with dH_2_O.

Purified peptide-protein inclusion bodies were solubilised with 6 mL of 85% formic acid and peptide released from the fusion by addition of 0.2 g cyanogen bromide, incubated in the dark at RT for 16–24 h. After incubation the formic acid solution was diluted with 20 mL of dH_2_O and lyophilised. Soluble peptides were then separated from the insoluble KSI stirring overnight in PBS (25 mM Na_2_HPO_4_, 100 mM NaCl; 2.5 mL per 400 mL original culture volume) the pH corrected to 7.5 and recovered by centrifugation. Peptide was then separated and purified from the contaminating His-tag using a Waters 600/486 series HPLC with a preparative Vidac C18 reverse phase protein and peptide column using an elution gradient of HPLC grade water and 70% acetonitrile/30% water containing 0.05% and 0.045% trifluoroacetic acid (TFA) respectively. Peptide containing fractions were collected when the absorbance of the flow at 220 nm reached 0.1 AU and stopped when the absorbance returned to 0.2 AU to minimise the risk of sample contamination. Collected fractions were then lyophilised to recover the peptide.

### NMR spectroscopy

All data were acquired at 10 °C using a 4-channel Varian UnityINOVA NMR spectrometer operating at 14.1 T (600 MHz ^1^H) with a 5 mm room temperature HCN probe. 2D ^13^C,^1^H-STD-HSQC experiments used a previously published pulse sequence,^14^ with Gaussian-based saturation^19^ at –3 ppm and –30 ppm. These experiments were acquired with 2048 data points (8000 Hz) in the direct F2 dimension and 64 data pairs – 128 points (18 000 Hz) in the indirect F1 dimension. ^13^C,^1^H-HSQC spectra for assignment were acquired with 512 complex pairs (1024 points) in F1 and each inversion recovery ^13^C,^1^H-HSQC dataset were acquired with 128 complex pairs (256 points) in F1; all with ^13^C spectral width of 18 000 Hz. In addition, inversion recovery experiments utilised a scan recycle delay of 5 seconds.

### STD and QSTD NMR analysis

Quantitative STD analysis utilises ^1^H longitudinal relaxation times (*T*_1_) of protons in each CH_*n*_ correlation to quantify 2D STD data. A comparison of Q2DSTD and 2DSTD data for all three peptides is shown in Fig. 1 and data for each resonance tabulated in ESI† observed. ^1^H *T*_1_ relaxation times were acquired using a modified ^13^C,^1^H-HSQC experiment with a ^1^H inversion-recovery segment (180°–*τ*–90°) in place of the initial 90° ^1^H-pulse as shown in the ESI.† The inter-sequence relaxation delay was set to 5 s and *τ* values were 0.2, 0.3, 0.4, 0.5, 0.6, 0.7, 0.8 and 1.0 s to provide intensities for each ^1^H resonance correlated to its ^13^C in the modified HSQC. Measurement of each series of intensities (*I*) for each ^1^H resonance enabled the determination of ^1^H *T*_1_ by curve-fitting the equation *I* = *I*_o_[1 – 2*e*(–*τ*/*T*_1_)]. Two-dimensional quantitative STD (Q2DSTD) for each ^1^H were obtained using the equations below with intensities from control (2DSTD_ctrl_) and difference (2DSTD_diff_) experiments^14^ where the ligand excess was 53-fold.2DSTD_amp_ = [2DSTD_diff_/2DSTD_ctrl_] × ligand excessQ2DSTD = 2DSTD_amp_/*T*_1_
