# Supplementary material for: Q2DSTD NMR deciphers epitope-mapping variability for peptide recognition of integrin αvβ6
Source: Org Biomol Chem. 2015 Jun 29;13(29):8001–7. doi: 10.1039/c5ob01237f (PMC4541471; doi:10.1039/c5ob01237f)
Supplement: Supplementary file 1 [file OB-013-C5OB01237F-s001.pdf]

Electronic Supplementary Material (ESI) for Organic & Biomolecular Chemistry.
This journal is © The Royal Society of Chemistry 2015

Q2DSTD NMR deciphers epitope-mapping variability for peptide recognition in integrin αvβ6

Jessica L. Sorge†, Jane L. Wagstaff†, Michelle L. Rowe, Richard A. Williamson*

and Mark J. Howard*

SUPPORTING INFORMATION

$^{1}H,^{13}C$-HSQC WITH INVERSION RECOVERY

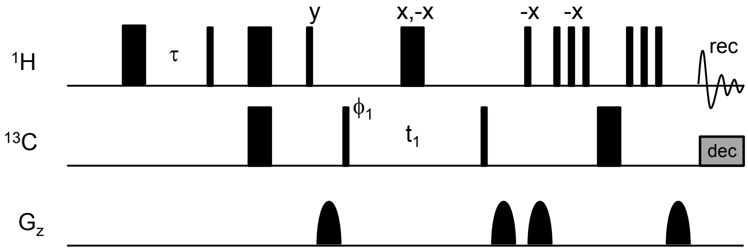

All phases are 'x' unless otherwise stated

ϕ_1 = x,-x (incremented for second dimension)

GARP ^{13}C decoupling was used during acquisition using Garp.

Receiver = x,-x,x,-x (control) or x,-x,-x,x (difference)

A comb of 'n' saturation pulses are applied over the required saturation period at the desired frequency offset to provide saturation transfer (on) or not (off).

Sat pulse = off,off,off,off (control)
= on,on,off,off (difference)

[180-τ-90] unit at the head of the sequence provides the adjustable element to measure T_1 of ^{1}H attached to ^{13}C. The inter-sequence relaxation delay was set to 5 s and τ values were 0.2, 0.3, 0.4, 0.5, 0.6, 0.7, 0.8 and 1.0 s to provide intensities for each ^{1}H resonance correlated to its ^{13}C in the modified HSQC and example intensity data are shown below for four ^{1}H-^{13}C correlations in FMDV2:

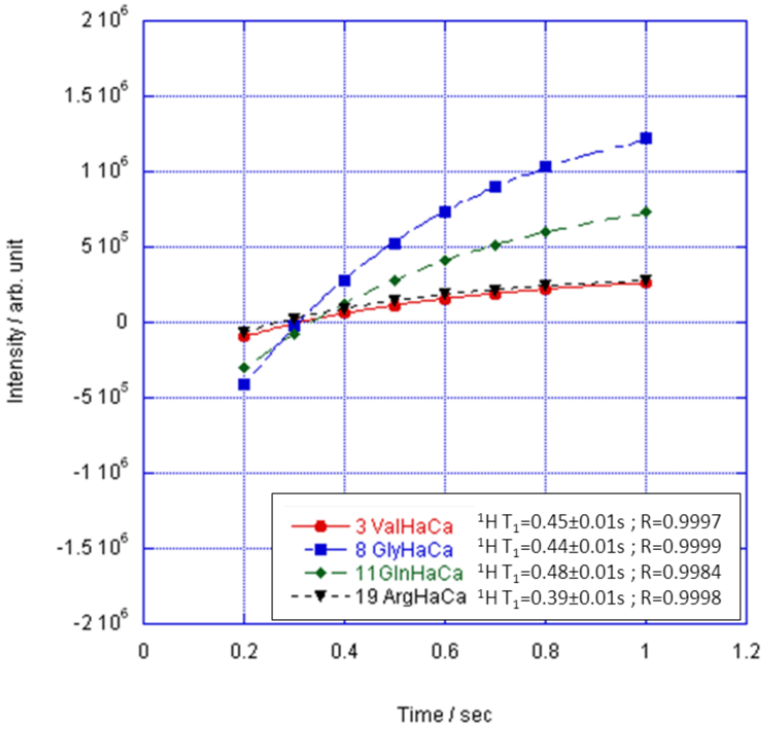

Curves were fitted to: $I_z = I_o [1 - 2e(-t/T_1)]$ where I_z is the measured intensity and t is the tau delay in 180-t-90 delay. Intensities were measured for each 2D dataset obtained with a specific tau value using CCPN Analysis.

PEPTIDE ^{1}H, ^{13}C HSQC ASSIGNMENTS

FMDV2

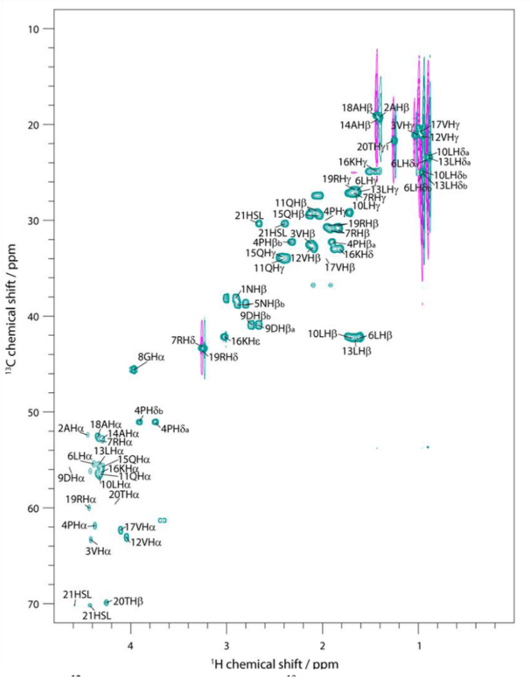

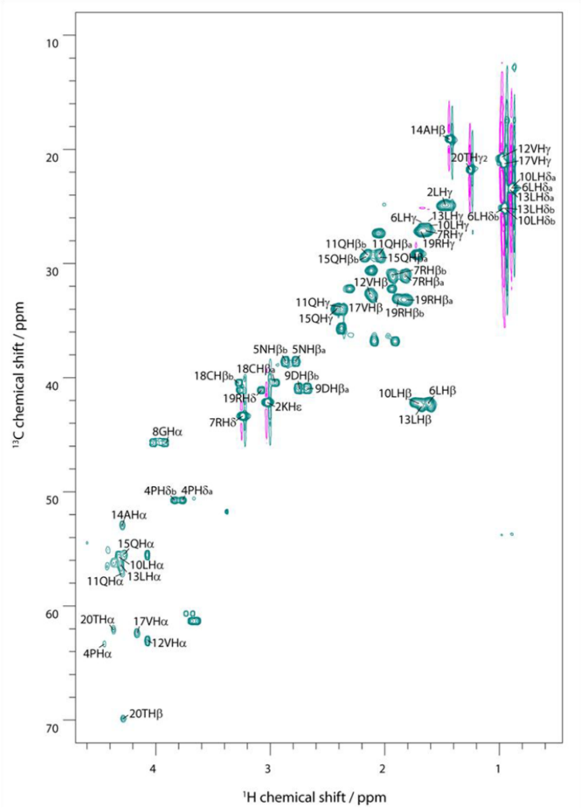
14AHβ
20THγ2
12VHγ
17VHγ
10LHδa
6LHδa
13LHδa
13LHδb
10LHδb
2LHγ
6LHγ
13LHγ
6LHδb
10LHγ
7RHγ
19RHγ
11QHβb
11QHβa
15QHβb
15QHβa
7RHβb
7RHβa
12VHβ
19RHβa
11QHγ
17VHβ
19RHβb
15QHγ
5NHβb
5NHβa
18CHβa
9DHβb
18CHβb
9DHβa
19RHδ
10LHβ
6LHβ
2KHε
7RHδ
13LHβ
8GHα
4PHδb
4PHδa
14AHα
15QHα
10LHα
11QHα
13LHα
20THα
17VHα
12VHα
4PHα
20THβ
¹³C chemical shift / ppm
¹H chemical shift / ppm

DBD1

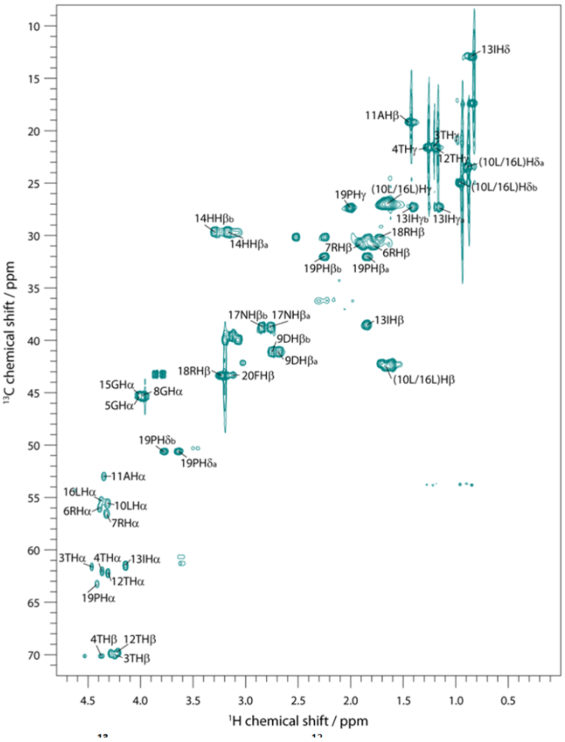
13IHδ
11AHβ
3THγ
4THγ
12THγ
(10L/16L)Hδa
(10L/16L)Hδb
19PHγ
(10L/16L)Hγ
13IHγb
13IHγa
14HHβb
14HHβa
18RHβ
7RHβ
6RHβ
19PHβb
19PHβa
17NHβb
17NHβa
9DHβb
9DHβa
13IHβ
18RHβ
20FHβ
(10L/16L)Hβ
15GHα
8GHα
5GHα
19PHδb
19PHδa
11AHα
16LHα
6RHα
10LHα
7RHα
3THα
4THα
13IHα
12THα
19PHα
4THβ
12THβ
3THβ
¹³C chemical shift / ppm
¹H chemical shift / ppm

LAP2T1

2DSTD, ^{1}H T_1 and Q2DSTD DATA.

Intensities are in arbitrary units and only for assignable peaks with measurable 2DSTD

FMDV2	2DSTD (arb units)	2DSTD %	^{1}H T_1 (s)	Q2DSTD (arb units)	Q2DSTD %
1 AsnHba			0.3358		
1 AsnHbb			0.3576		
2AlaHa			0.4223		
2AlaHb			0.3454		
3ValHa	1.7675	28.4	0.4503	3.9250	25.8
3ValHb	1.4695	23.6	0.4070	3.6109	23.7
3ValHga	1.5633	25.1	0.3826	4.0866	26.8
3ValHgb	1.0405	16.7	0.3073	3.3863	22.2
4ProHa	0.8140	13.1	0.4147	1.9631	12.9
4ProHb	0.9490	15.3	0.3856	2.4614	16.2
4ProHb	0.8269	13.3	0.3825	2.1621	14.2
4ProHd	0.1112	1.8	0.3868	0.2876	1.9
4ProHd	2.1186	34.1	0.3663	5.7842	38.0
4ProHg	1.5850	25.5	0.3500	4.5282	29.7
5AsnHb	0.7538	12.1	0.3563	2.1155	13.9
5AsnHb	0.8308	13.4	0.3505	2.3702	15.6
6LeuHa			0.4453		
6LeuHb	2.8360	45.6	0.3358	8.4444	55.5
6LeuHg	2.5494	41.0	0.3574	7.1334	46.8
6LeuHda	3.6967	59.5	0.3800	9.7288	63.9
6LeuHdb	3.1977	51.4	0.3453	9.2612	60.8
7ArgHa	3.4489	55.5	0.4720	7.3068	48.0
7ArgHb	1.3923	22.4	0.3047	4.5697	30.0
7ArgHb	1.4638	23.5	0.2902	5.0436	33.1
7ArgHd	0.5340	8.6	0.3183	1.6774	11.0
7ArgHg	1.6649	26.8	0.3527	4.7201	31.0
8GlyHa	1.7122	27.5	0.4448	3.8491	25.3
9AspHa			0.5460		
9AspHb	1.8762	30.2	0.3918	4.7884	31.4
9AspHb	2.4276	39.0	0.3947	6.1506	40.4
10LeuHa	1.7279	27.8	0.4635	3.7277	24.5
10LeuHb	2.6520	42.7	0.3266	8.1207	53.3
10LeuHg	2.7561	44.3	0.3590	7.6764	50.4
10LeuHda	3.6967	59.5	0.3660	10.1013	66.3
10LeuHdb	3.0376	48.9	0.3434	8.8445	58.1
11GlnHa	2.0364	32.8	0.4824	4.2218	27.7
11GlnHb	1.3477	21.7	0.3206	4.2042	27.6
11GlnHb	1.2536	20.2	0.3194	3.9243	25.8
11GlnHg	1.6858	27.1	0.3057	5.5147	36.2
12ValHa	1.4752	23.7	0.4976	2.9644	19.5
12ValHb	6.2168	100.0	0.4170	14.9084	97.9
12ValHga	4.4295	71.3	0.3619	12.2400	80.4

12ValHgb	3.0588	49.2	0.3576	8.5531	56.2
13LeuHa	0.8204	13.2	0.4677	1.7543	11.5
13LeuHb	2.5889	41.6	0.3393	7.6313	50.1
13LeuHg	5.2711	84.8	0.3461	15.2283	100.0
13LeuHda	3.6967	59.5	0.3773	9.7969	64.3
13LeuHdb	3.0376	48.9	0.3497	8.6864	57.0
14AlaHa	4.1979	67.5	0.5065	8.2879	54.4
14AlaHb	1.2396	19.9	0.3277	3.7831	24.8
15GlnHa	3.8847	62.5	0.5119	7.5883	49.8
15GlnHb	1.1015	17.7	0.3223	3.4176	22.4
15GlnHb	1.0499	16.9	0.3166	3.3158	21.8
15GlnHg	0.9749	15.7	0.3384	2.8807	18.9
16LysHa	0.6526	10.5	0.4420	1.4766	9.7
16LysHd	2.0962	33.7	0.3358	6.2428	41.0
16LysHe	0.3440	5.5	0.3810	0.9028	5.9
16LysHg			0.2884		
17ValHa	1.3796	22.2	0.4532	3.0445	20.0
17ValHb	2.2983	37.0	0.4014	5.7253	37.6
17ValHgb	1.9290	31.0	0.3777	5.1070	33.5
17ValHga	2.5163	40.5	0.3620	6.9503	45.6
18AlaHa	0.4732	7.6	0.4339	1.0907	7.2
18AlaHb	0.8604	13.8	0.3330	2.5835	17.0
19ArgHa			0.3902		
19ArgHb	2.1983	35.4	0.3055	7.1962	47.3
19ArgHb	1.2287	19.8	0.3093	3.9724	26.1
19ArgHd	0.5340	8.6	0.3025	1.7651	11.6
19ArgHg	2.5494	41.0	0.3696	6.8972	45.3
20ThrHaHa			0.4208		
20ThrHb	1.2814	20.6	0.4002	3.2021	21.0
20ThrHg2	0.7073	11.4	0.3250	2.1764	14.3

DBD1	2DSTD (arb units)	2DSTD %	$^{1}H\ T_1$ (s)	Q2DSTD (arb units)	Q2DSTD %
2LysHe	0.6356	13.7	0.3079	2.0641	17.2
2LysHg	0.5416	11.7	0.3748	1.4451	12.0
4ProHa	0.5177	11.2	0.6414	0.8072	6.7
4ProHda	3.6214	78.2	0.5481	6.6072	55.0
4ProHdb			0.5410		
5AsnHba	1.2575	27.1	0.4269	2.9457	24.5
5AsnHbb	0.4182	9.0	0.4302	0.9721	8.1
6LeuHb	3.6186	78.1	0.3861	9.3723	78.0
6LeuHda	3.7871	81.7	0.3855	9.8234	81.8
6LeuHdb	2.2018	47.5	0.3491	6.3072	52.5
6LeuHg	2.2122	47.7	0.4305	5.1392	42.8
7ArgHba	2.0666	44.6	0.3581	5.7711	48.0
7ArgHbb	1.3528	29.2	0.3596	3.7615	31.3
7ArgHd	0.8344	18.0	0.3841	2.1724	18.1
7ArgHg	2.3174	50.0	0.3275	7.0759	58.9
8GlyHa	3.1384	67.7	0.5215	6.0181	50.1
9AspHba	2.7270	58.8	0.4552	5.9903	49.9
9AspHbb	2.5512	55.1	0.4470	5.7081	47.5
10LeuHa	2.0312	43.8	0.5454	3.7245	31.0
10LeuHb	4.0946	88.4	0.3850	10.6349	88.5
10LeuHda	4.6339	100.0	0.3858	12.0126	100.0
10LeuHdb	3.5080	75.7	0.3480	10.0820	83.9
10LeuHg	2.4743	53.4	0.4219	5.8654	48.8
11GlnHa	1.0047	21.7	0.5429	1.8508	15.4
11GlnHba	2.3655	51.0	0.3759	6.2931	52.4
11GlnHbb	0.9782	21.1	0.3756	2.6041	21.7
11GlnHg	1.4832	32.0	0.3346	4.4330	36.9
12ValHa	4.0880	88.2	0.5828	7.0145	58.4
12ValHb	3.4079	73.5	0.4950	6.8850	57.3
12ValHga	3.2130	69.3	0.3744	8.5828	71.4
13LeuHa	1.4961	32.3	0.5569	2.6866	22.4
13LeuHb	3.1229	67.4	0.3925	7.9561	66.2
13LeuHda	3.4468	74.4	0.3855	8.9410	74.4
13LeuHdb	2.9676	64.0	0.3488	8.5087	70.8
13LeuHg	2.9533	63.7	0.4233	6.9776	58.1
14AlaHa	2.1391	46.2	0.5847	3.6581	30.5
14AlaHb	2.1278	45.9	0.3455	6.1596	51.3
15GlnHa	1.3481	29.1	0.4933	2.7330	22.8
15GlnHba	0.4361	9.4	0.3728	1.1697	9.7
15GlnHbb	1.0740	23.2	0.3748	2.8652	23.9
15GlnHg	0.8964	19.3	0.3429	2.6143	21.8
17ValHa	2.7131	58.5	0.5954	4.5566	37.9
17ValHb			0.4754		
17ValHga	2.6573	57.3	0.3397	7.8237	65.1
18CysHba	2.2176	47.9	0.5618	3.9473	32.9

18CysHbb	2.5545	55.1	0.5798	4.4059	36.7
19ArgHba	0.8893	19.2	0.3307	2.6893	22.4
19ArgHbb	2.6293	56.7	0.3428	7.6704	63.9
19ArgHd	4.0110	86.6	0.5928	6.7666	56.3
19ArgHg	1.9662	42.4	0.3855	5.1003	42.5
20ThrHa			0.4708		
20ThrHb	0.2921	6.3	0.4538	0.6438	5.4
20ThrHg2	0.8336	18.0	0.3333	2.5008	20.8

LAP2T1	2DSTD (arb units)	2DSTD %	$^{1}H\ T_1$ (s)	Q2DSTD (arb units)	Q2DSTD %
3ThrHa	0.0000		0.4059	0.0000	
3ThrHb	1.3159	26.9	0.3951	3.3306	34.1
3ThrHgb	1.5390	31.4	0.3261	4.7190	48.4
4ThrHa	1.2231	25.0	0.4436	2.7569	28.3
4ThrHb	2.1129	43.1	0.4071	5.1902	53.2
4ThrHgb	1.8330	37.4	0.3383	5.4179	55.5
5GlyHa	1.3554	27.7	0.3701	3.6619	37.5
6ArgHa	3.8886	79.4	0.4416	8.8049	90.3
6ArgHb	1.9230	39.3	0.3109	6.1858	63.4
6ArgHb	1.4149	28.9	0.3111	4.5487	46.6
7ArgHa	1.6649	34.0	0.4534	3.6717	37.6
7ArgHb	2.4377	49.8	0.3126	7.7980	79.9
7ArgHb	1.8437	37.6	0.3155	5.8431	59.9
8GlyHa	1.8870	38.5	0.4200	4.4927	46.1
9AspHb	1.9860	40.5	0.3851	5.1569	52.9
9AspHb	1.7975	36.7	0.3879	4.6342	47.5
(10Leu/16Leu)Hda	3.3187	67.7	0.3822	8.6839	89.0
(10Leu/16Leu)Hg	1.9889	40.6	0.3299	6.0280	61.8
(10Leu/16Leu)Hb	2.8272	57.7	0.3385	8.3517	85.6
(10Leu/16Leu)Hdb	2.9608	60.4	0.3552	8.3359	85.4
10LeuHa	1.6649	34.0	0.4660	3.5726	36.6
11AlaHa	2.2865	46.7	0.5096	4.4866	46.0
11AlaHb	1.9307	39.4	0.3346	5.7697	59.1
12ThrHa	2.3102	47.2	0.5028	4.5944	47.1
12ThrHb	0.9269	18.9	0.4424	2.0953	21.5
12ThrHgb	2.0515	41.9	0.3286	6.2425	64.0
13IleHa	3.2749	66.8	0.4695	6.9756	71.5
13IleHb	3.1528	64.4	0.4428	7.1204	73.0
13IleHda	4.8993	100.0	0.5924	8.2701	84.8
13IleHga	3.7093	75.7	0.3802	9.7555	100.0
13IleHgb	3.2227	65.8	0.3699	8.7135	89.3
14HisHb	2.7411	55.9	0.3803	7.2072	73.9
14HisHb	1.9431	39.7	0.3955	4.9132	50.4
15GlyHa	1.8196	37.1	0.3945	4.6125	47.3
16LeuHa	2.5773	52.6	0.4413	5.8408	59.9
(10Leu/16Leu)Hda	3.3187	67.7	0.3822	8.6839	89.0
(10Leu/16Leu)Hg	1.9889	40.6	0.3299	6.0280	61.8
(10Leu/16Leu)Hb	2.8272	57.7	0.3385	8.3517	85.6
(10Leu/16Leu)Hdb	2.9608	60.4	0.3552	8.3359	85.4
17AsnHb	1.9474	39.7	0.3383	5.7558	59.0
17AsnHb	1.9053	38.9	0.3380	5.6367	57.8
18ArgHb	2.2601	46.1	0.3130	7.2209	74.0
18ArgHb	1.4102	28.8	0.3124	4.5148	46.3
18ArgHd	1.2536	25.6	0.3052	4.1075	42.1
19ProHa	2.5304	51.6	0.4645	5.4473	55.8

19ProHb	2.7865	56.9	0.4065	6.8546	70.3
19ProHb	2.5513	52.1	0.4265	5.9817	61.3
19ProHd	3.7782	77.1	0.3910	9.6628	99.0
19ProHd	1.9892	40.6	0.3795	5.2413	53.7
19ProHg	3.5019	71.5	0.4539	7.7156	79.1
20PheHb	0.7835	16.0	0.2919	2.6840	27.5

Q2DSTD DATA SHOWN ACROSS EACH PEPTIDE SEQUENCE FOR ALL NUCLEI

Successive residues in sequence shown by alternating white then shaded backgrounds.

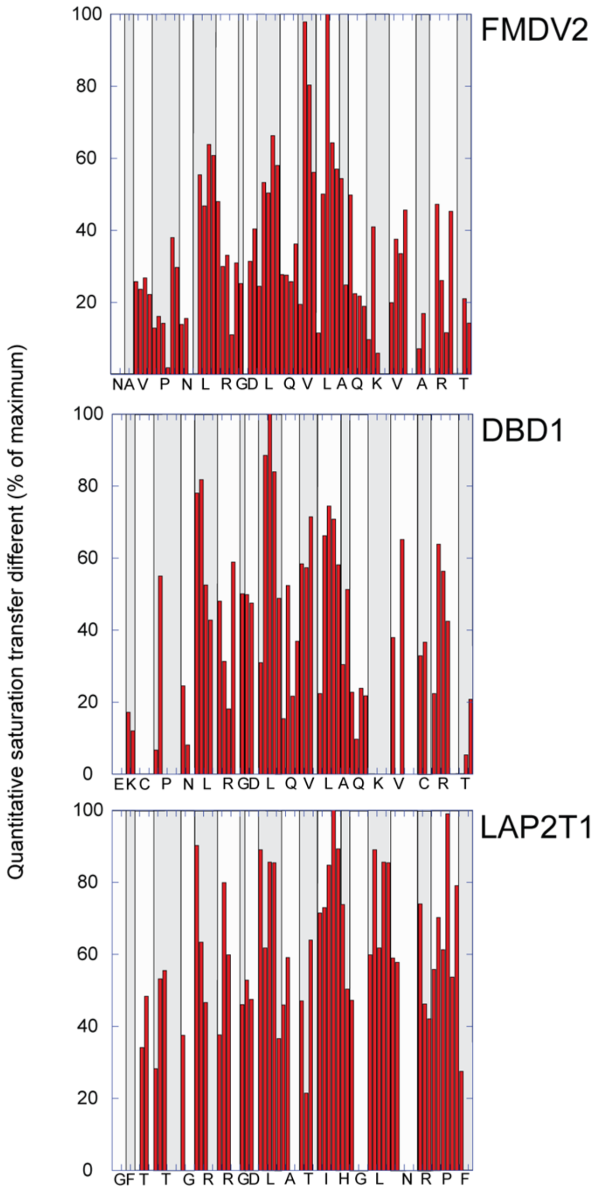

Note the general topology of FMDV2 and DBD2 are similar and this is to be expected because they are identical sequences (except for the disulphide cyclisation in DBD2).

However, the cyclisation appears to elevate N-terminal and C-terminal contacts in DBD1 as well as boost RGD-based contacts.

LAP projects significant contacts across the much of the peptide sequence and it is likely these contacts are also used when binding other integrins.

Chemical shifts (ppm) of $^{15}N/^{13}C$-A20fmdv2 in PBS

Residue	N	H^N	H^{α}	Others
1Asn			4.712	$H^{\beta2/\beta3}$ 2.866, 2.930; C^{β} 37.506;<br>$H^{\delta21/\delta22}$ 7.046, 7.766; $N^{\delta2}$ 112.751
2Ala	118.516	8.719	4.404	C^{α} 51.388; H^{β} 1.427; C^{β} 19.381
3Val	121.860	8.373	4.403	C’ 176.451; C^{α} 62.460; H^{β} 2.105; C^{β} 31.634;<br>$H^{\gamma1/\gamma2}$ 0.934, 1.055; $C^{\gamma1/\gamma2}$ 19.239, 20.190
4Pro				
5Asn	119.274	8.631	4.688	C’ 175.560; $H^{\beta2/\beta3}$ 2.813, 2.871; C^{β} 37.895;<br>$H^{\delta21/\delta22}$ 7.000, 7.708; $N^{\delta2}$ 112.940
6Leu	123.657	8.402<br>H^{γ} 1.594;	4.363	C’ 174.396; C^{α} 54.322; $H^{\beta2/\beta3}$ 1.696; C^{β} 41.311;<br>C^{γ} 25.713; $H^{\delta1/\delta2}$ 0.892, 0.935;<br>$C^{\delta1/\delta2}$ 22.572, 24.085
7Arg	121.294	8.427	4.313	C’ 176.604; C^{α} 52.200; $H^{\beta2/\beta3}$ 1.807, 1.931;<br>C^{β} 29.964; $H^{\gamma1/\gamma2}$ 1.654; C^{γ} 25.790;<br>$H^{\delta1/\delta2}$ 3.240; C^{δ} 42.295; H^{ε} 7.471; N^{ε} 117.442
8Gly	109.896	8.429	3.957	C’ 176.158; C^{α} 44.716
9Asp	120.457	8.392	4.584	C’ 173.182; C^{α} 53.552; $H^{\beta2/\beta3}$ 2.754; C^{β} 39.976
10Leu	121.952	8.224	4.303	C’ 175.904; C^{α} 55.541; $H^{\beta2/\beta3}$ 1.706; C^{β} 41.303;<br>H^{γ} 1.655; C^{γ} 26.054; $H^{\delta1/\delta2}$ 0.947, 0996;<br>$C^{\delta1/\delta2}$ 22.257, 23.916
11Gln	121.142	8.374	4.289	C’ 176.461; C^{α} 55.071; $H^{\beta2/\beta3}$ 2.032, 2.101;<br>C^{β} 28.222; $H^{\gamma1/\gamma2}$ 2.375; C^{γ} 32.936;<br>$H^{\varepsilon21/\varepsilon22}$ 6.937, 7.651; $N^{\varepsilon2}$ 112.990
12Val	121.838	8.146	4.024	C’ 175.458; C^{α} 62.189; H^{β} 2.088; C^{β} 31.828;<br>$H^{\gamma1/\gamma2}$ 0.921, 1.014; $C^{\gamma1/\gamma2}$ 19.553, 20.5668
13Leu	125.407	8.299	4.340	C’ 175.770; C^{α} 54.228; $H^{\beta2/\beta3}$ 1.682; C^{β} 41.279;<br>H^{γ} 1.600; C^{γ} 25.759; $H^{\delta1/\delta2}$ 0.895, 0.959;<br>$C^{\delta1/\delta2}$ 22.401, 23.991
14Ala	124.435	8.256	4.282	C’ 176.474; C^{α} 51.920; H^{β} 1.407; C^{β} 18.381
15Gln	119.394	8.284	4.284	C’ 177.137; C^{α} 54.693; $H^{\beta2/\beta3}$ 1.997, 2.123;<br>C^{β} 28.535; $H^{\gamma1/\gamma2}$ 2.416; C^{γ} 32.833;<br>$H^{\varepsilon21/\varepsilon22}$ 6.958, 7.631; $N^{\varepsilon2}$ 112.926
16Lys	123.025	8.379	4.289	C’ 175.310; C^{α} 55.156; $H^{\beta2/\beta3}$ 2.061, 2.365;<br>C^{β} 26.397; $H^{\gamma1/\gamma2}$ 1.445; C^{γ} 23.877;<br>$H^{\delta1/\delta2}$ 1.819; C^{δ} 31.972; $H^{\varepsilon2}$ 3.032; C^{ε} 41.159
17Val	121.959	8.221	4.091	C’ 175.666; C^{α} 61.405; H^{β} 2.084; C^{β} 17.969;<br>$H^{\gamma1/\gamma2}$ 0.918, 0.989; $C^{\gamma1/\gamma2}$ 19.558, 20.246
18Ala	128.359	8.467	4.406	C’ 175.041; C^{α} 51.471; H^{β} 1.412; C^{β} 19.252
19Arg	121.233	8.462	4.401	C’ 176.735; C^{α} 58.847; $H^{\beta2/\beta3}$ 1.804, 1.919;<br>C^{β} 29.617; $H^{\gamma1/\gamma2}$ 1.653; C^{γ} 25.306; $H^{\delta1/\delta2}$ 3.237;<br>C^{δ} 42.605; H^{ε} 7.243; N^{ε} 117.559
20Thr	115.687	8.300	4.394	C’ 175.650; C^{α} 58.899; H^{β} 4.306; C^{β} 69.058;<br>$H^{\gamma1}$ 1.244; C^{γ} 20.678
21Hsl	118.587	8.745	4.735	C’ 173.467; C^{α} 69.209; $H^{\beta2/\beta3}$ 2.376, 2.646;<br>C^{β} 29.328; $H^{\gamma1/\gamma2}$ 4.411, 4.588

Chemical shifts (ppm) of $^{15}N/^{13}C$-DBD1 in PBS

Residue	N	H^N	H^α	Others
1Glu				
2Lys	120.785	8.350	4.303	$H^{\beta2/\beta3}$ 1.856 1.904; $H^{\gamma1/\gamma2}$ 1.452; C^γ 23.892; $H^{\delta1/\delta2}$ 1.716; $H^{\varepsilon2}$ 3.046; C^ε 41.143
3Cys				
4Pro			4.461	$H^{\beta2/\beta3}$ 1.972, 2.325; H^γ 2.068; $H^{\delta1/\delta2}$ 3.786, 3.849; C^δ 49.656
5Asn	117.687	8.622	4.712	$H^{\beta2/\beta3}$ 2.796, 2.880; C^β 37.551; $H^{\delta21/\delta22}$ 7.076, 7.723; $N^{\delta2}$ 112.217
6Leu	122.229	8.321	4.438	C^α 41.251; $H^{\beta2/\beta3}$ 1.696; C^β 41.251; H^γ 1.631; C^γ 25.384; $H^{\delta1/\delta2}$ 0.902, 0.969; $C^{\delta1/\delta2}$ 22.357, 24.157
7Arg	120.235	8.445	4.381	$H^{\beta2/\beta3}$ 1.841, 1.950; C^β 30.003; $H^{\gamma1/\gamma2}$ 1.688; C^γ 26.385; $H^{\delta1/\delta2}$ 3.271; C^δ 42.350; H^ε 7.471; N^ε 115.791
8Gly	109.151	8.517	3.942	C^α 44.612
9Asp	119.439	8.388	4.612	$H^{\beta2/\beta3}$ 2.693, 2.765; C^β 39.229
10Leu	120.634	8.243	4.340	C^α 54.730; $H^{\beta2/\beta3}$ 1.753; C^β 41.208; H^γ 1.638; C^γ 25.731; $H^{\delta1/\delta2}$ 0.903, 0.978; $C^{\delta1/\delta2}$ 22.225, 24.090
11Gln	119.416	8.385	4.321	C^α 56.098; $H^{\beta2/\beta3}$ 2.066, 2.146; C^β 28.155; $H^{\gamma1/\gamma2}$ 2.405; $H^{\varepsilon21/\varepsilon22}$ 6.964, 7.682; $N^{\varepsilon2}$ 111.927
12Val	119.341	8.078	4.086	C^α 62.038; H^β 2.150; C^β 31.886; $H^{\gamma1/\gamma2}$ 0.989; $C^{\gamma1/\gamma2}$ 19.847
13Leu	123.098	8.260	4.328	C^α 55.135; $H^{\beta2/\beta3}$ 1.726; C^β 41.338; H^γ 1.636; C^γ 25.531; $H^{\delta1/\delta2}$ 0.913, 0.982; $C^{\delta1/\delta2}$ 22.484, 24.102
14Ala	122.829	8.207	4.306	C^α 51.927; H^β 1.447; C^β 18.063
15Gln	117.523	8.275	4.306	C^α 54.248; $H^{\beta2/\beta3}$ 2.059, 2.179; C^β 28.552; $H^{\gamma1/\gamma2}$ 2.437; C^γ 33.222; $H^{\varepsilon21/\varepsilon22}$ 7.007, 7.655; $N^{\varepsilon2}$ 111.911
16Lys	121.947	8.587	4.758	
17Val	118.487	8.124	4.176	C^α 61.332; H^β 2.127; C^β 31.916; $H^{\gamma1/\gamma2}$ 0.984; $C^{\gamma1}$ 20.079
18Cys	121.045	8.899	4.786	$H^{\beta2/\beta3}$ 2.965, 3.282; C^β 32.195
19Arg	122.899	8.597	4.511	$H^{\beta2/\beta3}$ 1.842, 1.932; C^β 32.195; $H^{\gamma1/\gamma2}$ 1.687; C^γ 26.326 H^ε 7.307; N^ε 115.904
20Thr	115.075	8.383	4.380	C^α 61.152; H^β 4.257; C^β 68.848; $H^{\gamma1}$ 1.270; C^γ 20.750
21Hsl	117.642	8.768	4.726	$H^{\beta2/\beta3}$ 2.405, 2.267; $H^{\gamma1/\gamma2}$ 4.434, 4.587

Chemical shifts (ppm) of $^{15}N^{13}C$-A20LAP2T1

Residue	N	H^N	H^α	Others
1Gly				
2Phe				
3Thr	115.931	8.435	4.475	C^α 61.606; H^β 4.255; C^β 70.151; $H^{\gamma1}$ 1.231; C^γ 21.475
4Thr	115.525	8.308	4.377	C^α 62.032; H^β 4.299; C^β 69.874; $H^{\gamma1}$ 1.282; C^γ 21.553
5Gly	110.541	8.561	4.022	C^α 45.373
6Arg	120.226	8.394	4.398	C^α 56.019; $H^{\beta2/\beta3}$ 1.791, 1.888; C^β 30.986; H^γ 1.655; $H^{\delta1/\delta2}$ 3.230; H^ε 7.321; N^ε 116.594
7Arg	122.369	8.662	4.331	C^α 56.612; $H^{\beta2/\beta3}$ 1.842, 1.917; C^β 32.638; $H^{\gamma1/\gamma2}$ 1.693; $H^{\delta1/\delta2}$ 3.240; H^ε 7.405; N^ε 116.482
8Gly	109.453	8.603	3.976	C^α 45.325
9Asp	119.548	8.324	4.637	$H^{\beta2/\beta3}$ 2.701, 2.755; C^β 41.067
10Leu	121.550	8.298	4.330	C^α 55.472; $H^{\beta2/\beta3}$ 1.707; C^β 42.416; H^γ 1.627; C^γ 26.950; $H^{\delta1/\delta2}$ 0.913, 0.971; $C^{\delta1/\delta2}$ 23.380,24.948
11Ala	123.034	8.374	4.359	C^α 52.956; H^β 1.450; C^β 19.115
12Thr	112.477	8.108	4.319	C^α 62.196; H^β 4.221; C^β 69.575; $H^{\gamma1}$ 1.196; C^γ 21.643
13Ile	121.950	8.146	4.153	C^α 61.460; H^β 1.862; C^β 38.495; $H^{\gamma12/\gamma13}$ 1.174, 1.417; C^γ 27.276; C^δ 12.907
14His	121.910	8.524	4.730	$H^{\beta2/\beta3}$ 3.153, 3.266; C^β 29.621
15Gly	109.081	8.454	3.987	C^α 45.102
16Leu	120.661	8.298	4.375	C^α 55.259; $H^{\beta2/\beta3}$ 1.700; C^β 42.416; H^γ 1.625; C^γ 26.950; $H^{\delta1/\delta2}$ 0.913, 0.971; $C^{\delta1/\delta2}$ 23.380, 24.948
17Asn	118.394	8.614	4.715	$H^{\beta2/\beta3}$ 2.773, 2.852; C^β 38.726; $H^{\delta21/\delta22}$ 7.027, 7.711; $N^{\delta2}$ 118.394
18Arg	121.252	8.282	4.652	$H^{\beta2/\beta3}$ 1.738, 1.850; C^β 30.274; $H^{\gamma1/\gamma2}$ 1.668; $H^{\delta1/\delta2}$ 3.237; C^δ 43.334; H^ε 7.258; N^ε 116.687
19Pro			4.425	C^α 63.194; $H^{\beta2/\beta3}$ 1.848, 2.267; C^β 32.020; $H^{\gamma1/\gamma2}$ 2.022; C^γ 27.324; $H^{\delta1/\delta2}$ 3.651, 3.785; C^δ 50.579
20Phe	119.376	8.456	4.601	$H^{\beta2/\beta3}$ 3.146; C^β 43.272
21Hsl	117.115	8.567	4.357	$H^{\beta2/\beta3}$ 2.264, 2.529; $H^{\gamma1/\gamma2}$ 4.383
